# The importance of acknowledging statisticians as named authors

**DOI:** 10.1007/s00417-020-04670-3

**Published:** 2020-04-15

**Authors:** Reinhard Vonthein, Catey Bunce, Diana Epstein, Paul Henry John Donachie

**Affiliations:** 1 Institut für Medizinische Biometrie und Statistik, Universität zu Lübeck, Universitätsklinikum Schleswig-Holstein, Campus Lübeck, Ratzeburger Allee 160, 23538 Lübeck, Deutschland Germany; 2 School of Population Health and Environmental Sciences, Faculty of Life Sciences and Medicine, King’s College London, 4th Floor, Addison House, Guy’s Campus, London, SE1 1UL UK; 3 Editorial Office, Graefe’s Archive for Clinical and Experimental Ophthalmology, Di-Ep Biomedical Editorial Services Ltd, PO Box 5617, Glasgow, Scotland G77-9EH UK; 4 The Royal College of Ophthalmologists National Ophthalmology Database, Gloucestershire Retinal Research Group office, Above Oakley Ward, Cheltenham General Hospital, Cheltenham, GL53 7AN UK

Many papers published in the field of Clinical and Experimental Ophthalmology use statistical methods, hence the importance of statisticians on the editorial board of our journal Graefe’s Archive for Clinical and Experimental Ophthalmology.

Our responsibility is focused on ensuring that methods are reproducible, the analysis is appropriate, and the results are useful (e.g., reporting standard deviations for use with planning future studies). Advanced statistical methods are rarely applied, and perhaps one reason for this is the suggestion that statisticians cannot be named authors or a restriction upon the number of statisticians that may be included as co-authors. In the editorial published in 2011 [1], it was noted: “In Graefe’s, a statistician can be a named author.” We would now wish to go further than this and encourage authors to engage statisticians where possible.

What would happen if statisticians were acknowledged as named authors?

First of all, we believe that there would be an increase in the use of appropriate statistical methods. One of the most frequent reasons for paper rejection is suboptimal or even incorrect statistics. In our journal, a total of 76 papers have been sent for statistical peer review during the past 2 years (2018 and 2019), 40 papers were accepted, 26 papers rejected, and 13 papers received the option to transfer to alternate journals since the authors were unable to revise.

An author contributes according to the following current definitions, by the International Committee of Medical Journal Editors [2] all of:

- Acquisition, analysis, or interpretation of data,
- Drafting the paper or revising the content,
- Final approval of the work,
- Responsibility, integrity, and accountability.

Statisticians who are collaborating with you will meet the criteria by:

- Helping design the study, advising on appropriate data collection, and taking responsibility for statistical analysis—descriptive (graphs) and inferential—the time to consult a statistician is before data collection wherever possible,
- Translating statistical methodology used in the analysis—so that statistical terminology is understood by research collaborators,
- Identifying limitations with the data and analysis and ensuring interpretation of analyses are robust, being responsible for statistical analyses and more [3],
- Authors respond in full to statistical reviewers’ comments.

The research project has more chance of proceeding well if all collaborators agree to the project plan beforehand. Many published articles need the contribution of specialists from more than one field. The benefit of reporting any statistics is obvious (see (a) above). Papers with a lack of statistics (b) or poorly integrated (c) may not be sent for external peer review. Acceptance for publication is more likely, when all specialists in the team respond to review comments (d). Are there downsides?

There may be a cost. Statisticians, like other professionals, need resourcing. You may find the statistician irritating—their tendency to stay on the fence and temper conclusions may jar. A classification for statistical co-authorship is described in greater detail in *Statistics in Medicine* [4].

Some research may necessitate the use of advanced statistics, and some projects can be lengthy; thus, more than one statistician may be required. Statisticians usually work in departments distinct from ophthalmology but with a similar inner structure and pressure to publish. Many projects could profit from a formal collaboration with two or more statisticians working on different parts of the project―e.g., preparatory work and analysis of final results―as a result being acknowledged as a named co-author in one article. In our own experience, the papers worked in collaboration with ophthalmologists, and one or two statisticians have resulted in higher citations and often higher than the journals’ impact factors suggested [5]. The Journal of the American Medical Association even required analyses by independent statisticians from 2005 to 2013 [6]. Collaborations with more than one statistician may spark statistics papers with ophthalmologists as co-authors.

Peer review by statisticians has led to recommendations like the CONSORT-Statement [7]. Our presence on this journal’s editorial board makes this visible. Please reciprocate the transparency. The joint work is important for validity and credibility.

We would like to take this opportunity and thank our statistical reviewers who help and support the journal with the peer review of papers. This helps in upholding the standard of the journal, and your assistance is very much appreciated.
